# Hæmaturia and Nephritis of Sudden Onset as an Initial Symptom of Typhoid Fever

**Published:** 1911-02-25

**Authors:** 


### HÆMATURIA AND NEPHRITIS OF SUDDEN ONSET AS AN INITIAL SYMPTOM OF TYPHOID FEVER.

The onset of typhoid fever is, as a rule, gradual and obscure, but in certain instances the malady begins acutely and presents symptoms which for the time being seem to indicate an entirely different disease. One is familiar, for instance, with cases in which the first symptoms seem to be in the lungs, and lobar-pneumonia is the first diagnosis made. In other instances the part of the bowel which first becomes inflamed is the vermiform appendix, and there may even be an appendicular abscess for which laparotomy is performed. Less common than either of these, and yet very misleading when met with, is acute nephritis as an early symptom in typhoid fever, such as has been recorded recently in more than one case in France.

In one instance a young man of eighteen years of age came to hospital suffering from hæmaturia, which had begun acutely eight days previously after exposure to severe cold. The urine was normal in amount, contained a large amount of albumin, and

. s of a dark reddish-black colour; microscopically he sediment exhibited granular tube casts and many deformed red corpuscles. Bacteriological cultivations were made both from the patient's blood and from his urine, and these remained negative. The other symptoms in the case were intense headache, a dicrotic pulse beating at about 100 per minute, a temperature of 104° F., bronchitic râles scattered over both lungs, some hypertrophy of the spleen, a tendency to constipation, and finally a decided œdema of the subcutaneous tissue, particularly in the lower limbs.

Looking back at this case, the dicrotion of the pulse and its relative slowness in proportion to the temperature, together with the palpability of the spleen, seem clearly enough to indicate that typhoid fever should have been thought of on admission; but so definite were both the œdema and the hæmaturia that it was not until eight days later that enterica was recognised as the result of the development of rose-red spots upon the abdomen. Five days later this was confirmed by Widal's test. Till this time the urine continued to contain an abundance of blood, but after the first ten days of the patient's stay in hospital both the blood and the albumin began to diminish, and at the end of four weeks, when the temperature had finally dropped to normal by lysis, the urine was perfectly clear. Polyuria succeeded this for a time, and even on the patient's discharge the urine contained 0.25 part of albumin per 1000.

The course of the disease seemed to be what one might term "average," the prognosis being rendered in no way worse by the fact that acute nephritis constituted so early and prominent a complication.

